# A patient with granulomatous amoebic encephalitis caused by *Balamuthia mandrillaris* survived with two excisions and medication

**DOI:** 10.1186/s12879-021-07020-8

**Published:** 2022-01-15

**Authors:** Limei Peng, Quan Zhou, Yu Wu, Xiaoli Cao, Zili Lv, Minghua Su, Yachun Yu, Wen Huang

**Affiliations:** 1 Department of Neurology, The First Affiliated Hospital of Guangxi Medical University, Nanning, 530021 China; 2 Department of Neurosurgery, The First Affiliated Hospital of Guangxi Medical University, Nanning, 530021 China; 3 Department of Neuroscience, University of Pittsburgh, 15262 Pittsburgh, PA USA; 4 Department of Pathology, The First Affiliated Hospital of Guangxi Medical University, 530021 Nanning, China; 5 Department of Infection, The First Affiliated Hospital of Guangxi Medical University, 530021 Nanning, China

**Keywords:** Granulomatous amoebic encephalitis, *Balamuthia mandrillaris*, High-throughput next-generation sequencing, Treatment

## Abstract

**Background:**

Granulomatous amoebic encephalitis (GAE) is a rare central nervous system infection caused by the *Balamuthia mandrillaris* or *Acanthamoeba* species. Diagnosis is challenging because of the non-specific clinical presentation, cerebrospinal fluid analysis, and radiological features. There is no effective treatment for GAE to date.

**Case presentation:**

A 54-year-old male was admitted to hospital after experiencing acute onset of numbness and weakness on his left limb. Due to the initial consideration of intracranial tumor, surgical removal of the right parietal lesion was performed. However, the patient had a headache accompanied by diplopia, difficulty walking and a new lesion was found in the left occipital-parietal lobe two weeks after the first operation. High-throughput next-generation sequencing (NGS) detected the presence of high copy reads of the *B. mandrillaris* genome sequence in the patient’s blood, cerebral spinal fluid (CSF), and brain tissue. Pathological investigation of the brain tissue showed granulomatous changes and amoebic trophozoite scattered around blood vessels under high magnification. The patient was re-operated due to developing progressive confusion caused by subfalcine herniation of the left cerebral hemisphere. The lesions of the right parietal lobe were obviously decreasing in size after the first surgery, and the lesions of the left occipital lobe and the sunfalcine herniation didn’t ameliorate two months after the second surgery. The patient was transferred to local hospital for continuous treatment with sulfamethoxazole and azithromycin. After five months of the second surgery, the patient showed good recovery with mild headache.

**Conclusions:**

This is the first report of a patient with *B. mandrillaris* encephalitis initially confirmed by NGS and have experienced two excisions, responding favorably to the combination of surgeries and medications. Early surgical resection of intracranial lesions combined with drug treatment may offer the chance of a cure.

**Supplementary Information:**

The online version contains supplementary material available at 10.1186/s12879-021-07020-8.

## Background

Granulomatous amoebic encephalitis (GAE) is a rare central nervous system (CNS) infection caused by *Balamuthia mandrillaris* or *Acanthamoeba* species; the infection progresses chronically or subacutely, during a period of few weeks to few years. *B. mandrillaris* is a free-living species of amoebae which was first isolated in 1989 [1]. It exists in water or soil, and infects humans through the skin or respiratory tract. It may also infect humans through organ transplantations by spreading through blood, or through direct invasions in the CNS through the olfactory neuroepithelium [2, 3]. *B. mandrillaris* encephalitis (BAE) has been predominantly found in South America and the United States. Only few cases have been reported in China. The onset symptom of GAE is insidious and followed by rapid severe neurological decline, including headaches, seizures, altered levels of consciousness, comas, and death. Diagnosis is challenging because of the non-specific clinical presentation, cerebrospinal fluid (CSF) analysis, and radiological features.

Here, we describe a middle-aged male patient from Southern China, who was diagnosed with *B. mandrillaris* encephalitis confirmed by High-throughput next-generation sequencing (NGS), and survived with two excisions and medications (Additional file 1).

## Case presentation

**Fig. 1:**
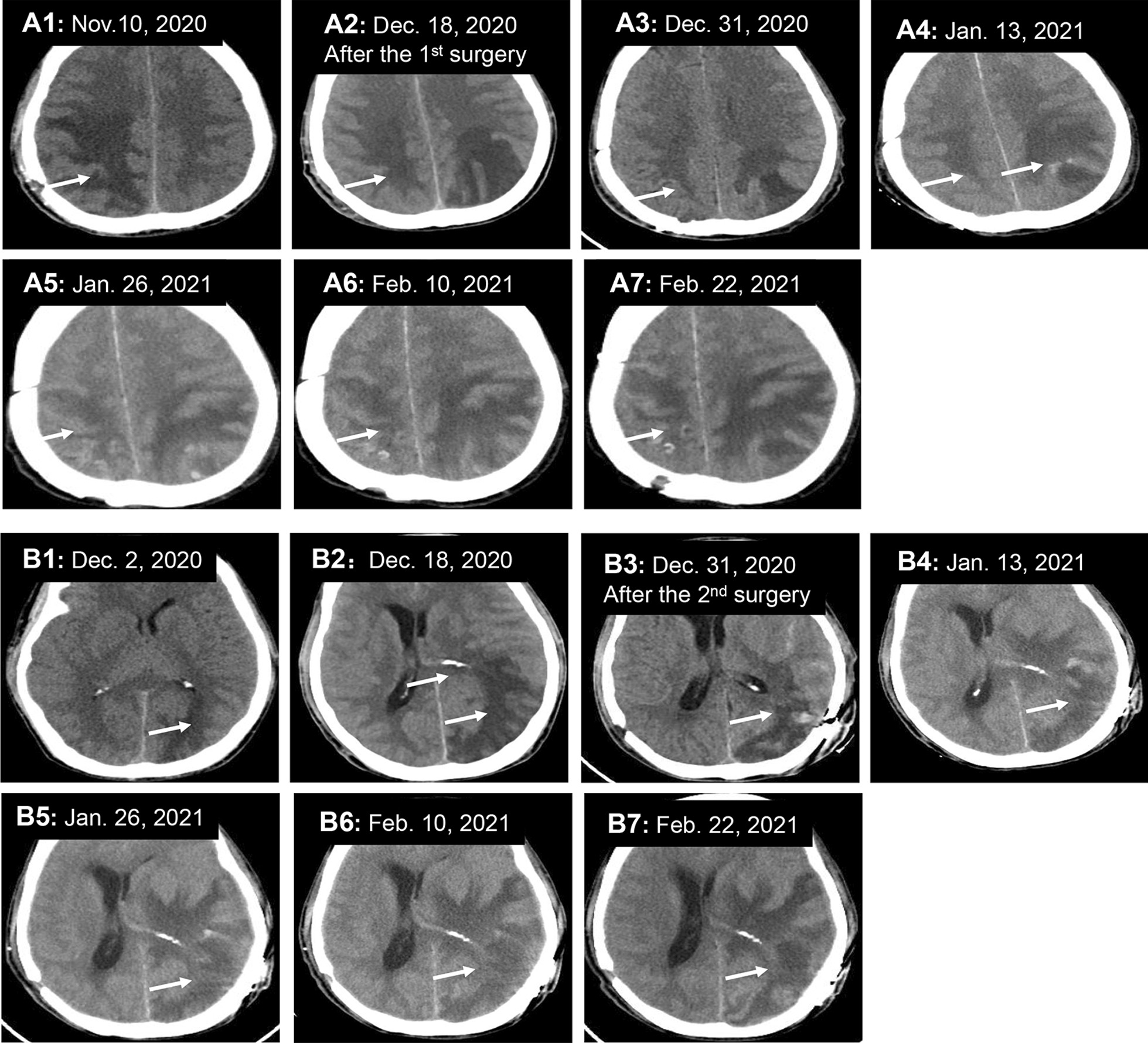
The dynamic changes of head CT. **A** The original lesion in the right parietal lobe was getting smaller; **B** a new lesion occurring in the left occipital lobe became worse

**Fig. 2:**
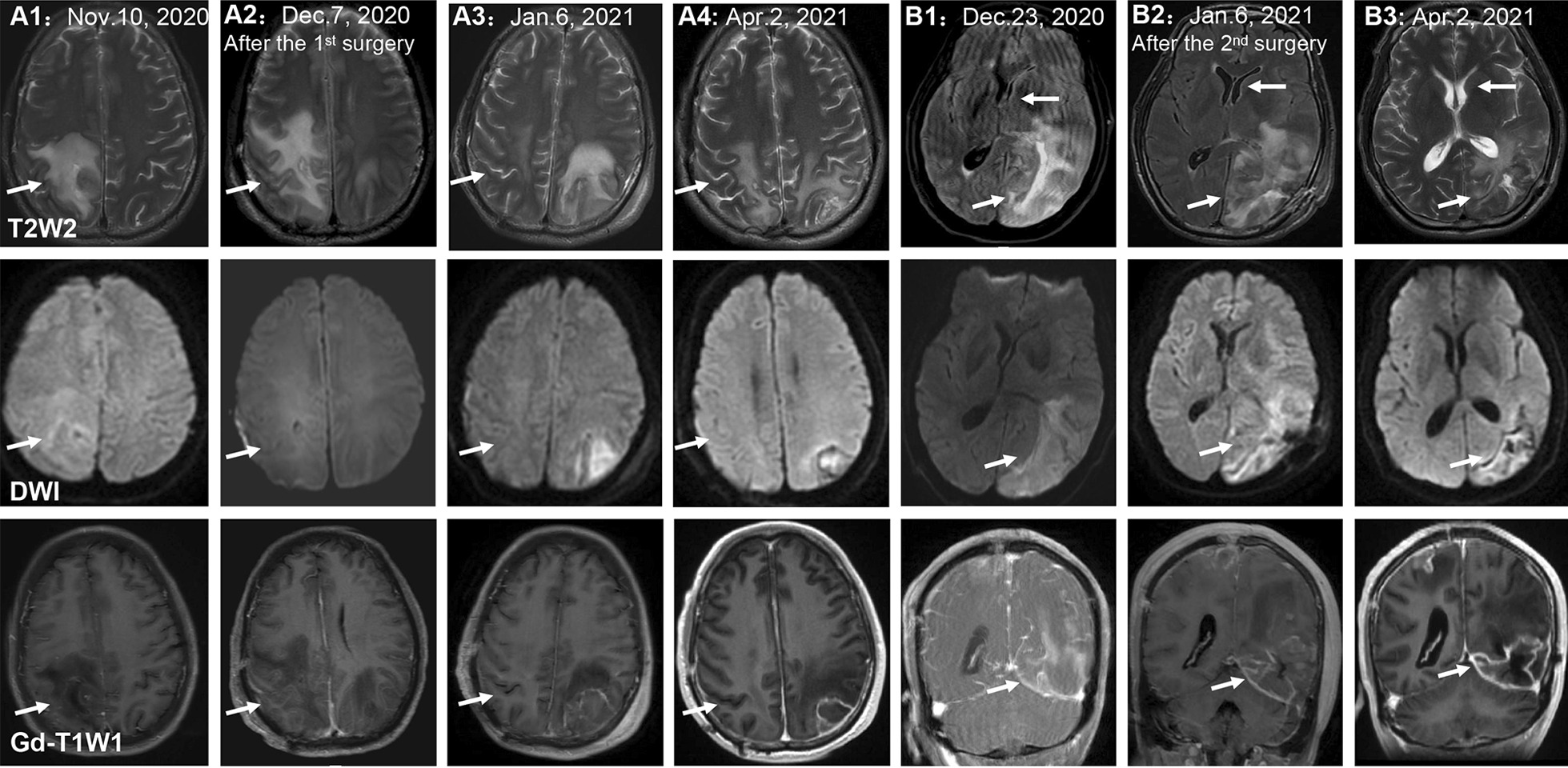
The dynamic changes of brain magnetic resonance imaging (MRI). **A1–A4** The lesions of the right parietal lobe were obviously getting smaller on T2-weighted image (T2W2). **B1**–**B3** The lesions of the left occipital lobe and the left cerebral subfalcine herniation became better slightly. Both lesions of the right parietal lobe and the left occipital lobe showed high signal intensity on T2W2, obvious high signal intensity on diffusion-weighted image (DWI) and enhancement on gadolinium-enhanced T1-weighted image (Gd-T1WI) in the left occipital lobe lesions, milder enhancement in the right parietal lobe

**Fig. 3:**
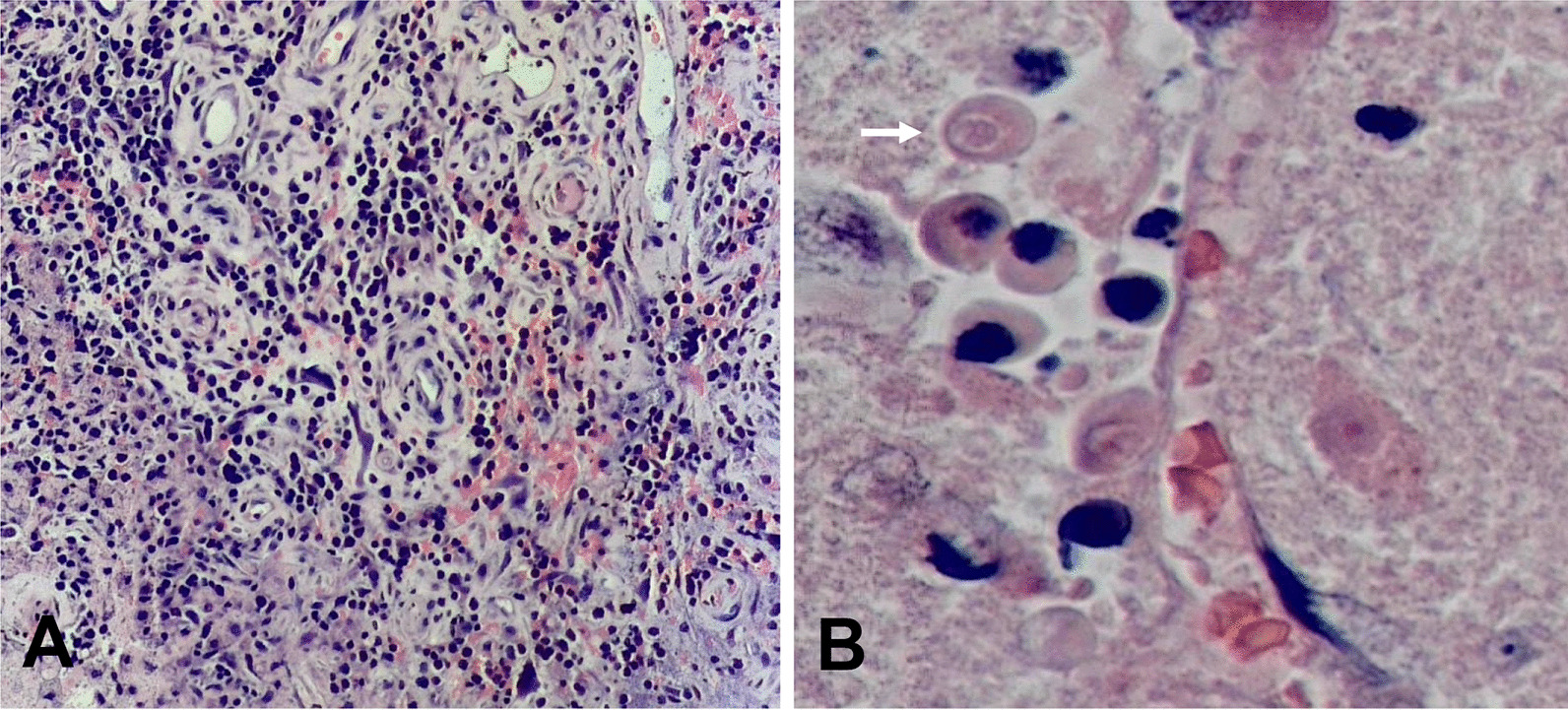
Histological examinations. **A** Granulomatous changes and inflammatory perivascular infiltrate (×100); **B** Amoea trophozoite (×400)

**Fig. 4:**
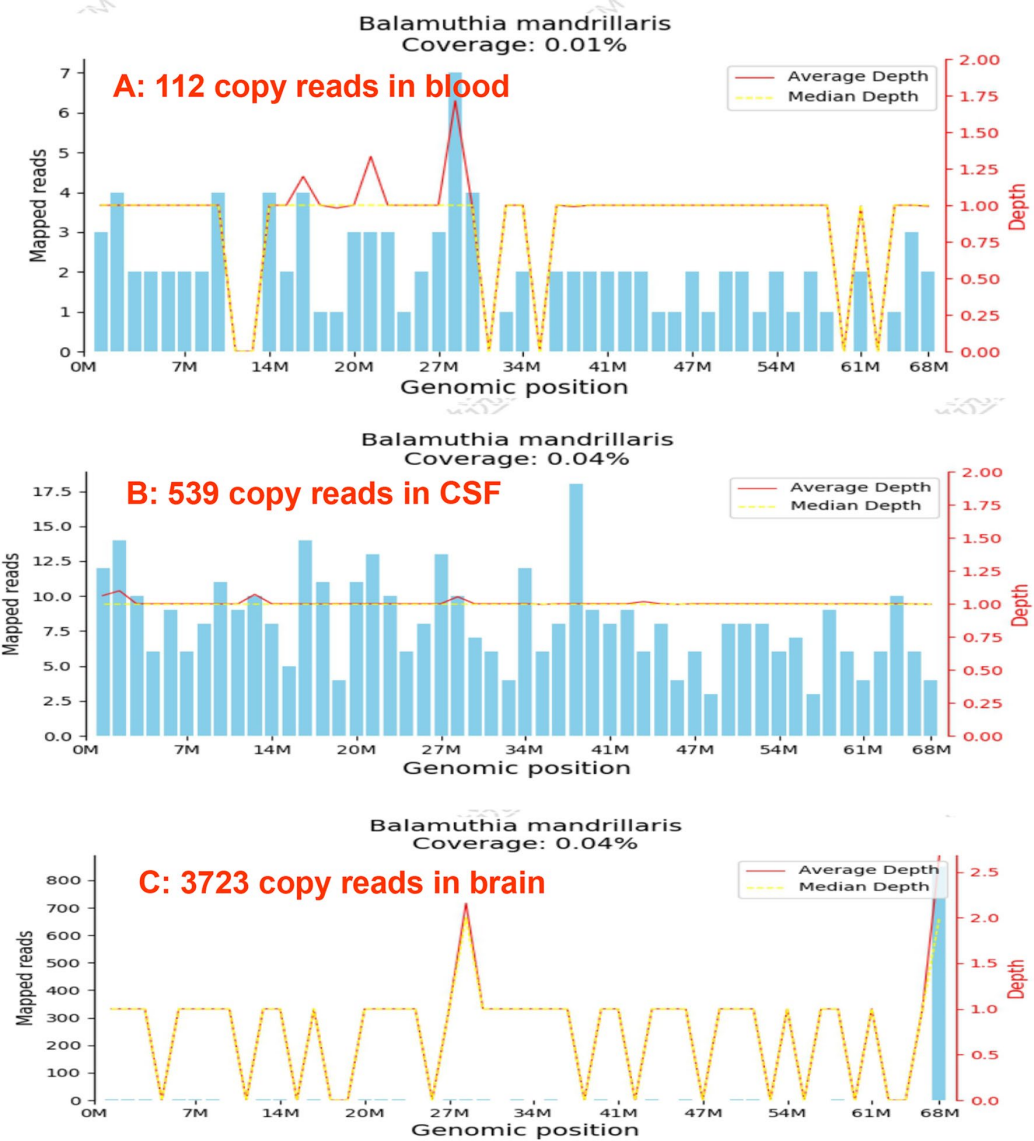
The results of high-throughput next-generation sequencing (NGS). Balamuthia mandrillaris with 112 sequence copy reads in serum (**A**), 539 sequence copy reads in CSF (**B**), and 3723 sequence copy reads in brain tissue (**C**)

A 54-year-old male experiencing acute onset of numbness and weakness in his left limb was admitted to the First Affiliated Hospital of Guangxi Medical University on Nov. 1, 2020. Initial magnetic resonance imaging (MRI) of the brain showed abnormal signals in the right parietal lobe. Brain computed tomography (CT) (Fig. 1A1) and enhanced MRI (Fig. 2A1) on Nov. 10 showed an ill-defined infiltrating enhanced mass in the right parietal lobe with perilesional edema, which pointed to the possibility of a low-grade glioma. Resection of the mass in the right parietal lobe was performed on Nov. 12. The specimen exhibited granulomatous changes and inflammatory perivascular infiltrate (Fig. 3A) with positive CD68 staining, negative acid-fast, periodic acid-achiff and CD235a staining. The patient consequently received antibiotic therapy: intravenous Linezolid (0.2 g twice daily for 9 days). His left limb weakness improved slightly, as seen in his activity of daily living (ADL) scores. The ADL score was 75 after the surgery in comparison to 70 before the surgery. However, the patient had a headache accompanied by diplopia and difficulty walking when he was taking hyperbaric oxygen therapy on Nov. 28. An emergency head CT on Dec. 2 showed that a new low-density focus occurred in the left occipital lobe (Fig. 1B1). The blood tests on Dec. 5 revealed that erythrocyte sedimentation rate was 31 mm/h (0–15), hypersensitive C-reactive protein was 0.8 mg/L (0-1), immunoglobulin G subtype 4 was 4.04 g/L and antinuclear antibody was weakly positive. The white blood cells, neutrophils, lymphocytes, eosinophils and platelets were in normal range except for a slight decrease of red blood cells and hemoglobin level. The other tests including the vasculitis antibodies, fungal (1-3)-β-D glycan, and galaetomannan test were normal. Brain enhanced MRI on Dec.7 showed a new abnormal signal focus in the left occipital-parietal lobe, and the operation area of the right parietal lobe had more obvious effusion and edema (Fig. 2A2). Lumbar puncture on Dec. 7 revealed high intracranial pressure (330 mmH_2_O), elevation in white cell count (320*10^6^/L, neutrophils 45%, lymphocytes 55%) and protein (1041 mg/L), as well as a slight decrease in glucose (2.01 mmol/L) and chloride (116.1 mmol/L). No acid fast bacilli, fungi and bacteria were found in the cerebrospinal fluid (CSF) smear and culture. Despite a treatment with intravenous Ceftriaxone Sodium (2 g twice daily for 4 days), the patient still had an obvious headache, diplopia, non-fluent speech and worse muscle strength in his left limbs. Neurological examination on Dec. 10 showed left eyeball adduction, left eyeball abduction, and right eyeball abduction were impaired. Re-examination of lumbar puncture on Dec.11 showed that the CSF data were getting worse (intracranial pressure 330 mmH_2_O, white cell count 480*10^6^/L with neutrophils 40%, lymphocytes 60% and protein 1422 mg/L, glucose 1.24 mmol/L and chloride 111.5 mmol/L). Intravenous Vancomycin (1.0 g iv drip Q12h) and Meropenem (0.5 g iv drip Q6h) were applied on Dec.11 due to consideration of intracranial infection caused by bacteria. High-throughput next-generation sequencing (Vision Medicals, Guangzhou) detected the presence of *Balamuthia mandrillaris* with 112 sequence copy reads in serum and 539 sequence copy reads in the CSF (Fig. 4A, B). GAE caused by *Balamuthia mandrillaris* was thus diagnosed. On Dec.14, the treatment was adjusted to compound treatment with sulfamethoxazole/trimethoprim 0.48 g×2 tablets po. Bid, azithromycin tablets 0.25 g×2 po. Qd, flucytosine 2.5 g iv drip Q12 h, and amphotericin B iv drip Qd (increased gradually with 5–10–15–20–25–30 mg). He did not receive a combination therapy of miltefosine and pentamidine because neither medication was available in China. The patient often went fishing in the past 10 years and worked occasionally in a paddy field. He had no known tick bites or any skin lesions.

Re-examination of brain MRI on Dec. 23 showed larger lesions on the left cerebral hemisphere with enhancement, causing left subfalcine herniation (Fig. 2B1). The patient was transferred to the department of neurosurgery and was re-operated on Dec. 27. Histological examination of the left occipital lobe confirmed amoebic trophozoite gathering around blood vessels under high magnification (Fig. 3B). The presence of *B. mandrillaris* with 3723 sequence copy reads was also detected in brain tissue via NGS (Fig. 4C). After surgery, re-examination of brain MRI on Jan. 6, 2021 indicated that the lesion and subfalcine herniation of the left cerebral hemisphere were relieved slightly than before (Fig. 2B2). However, the patient developed progressive confusion. A repeated head CT on Jan. 13 revealed that the left subfalcine herniation became worse (Fig. 1B4) after medication therapy was discontinued for two weeks. A combined medication therapy was restarted with sulfamethoxazole/trimethoprim (0.48 g×2 po. Bid), azithromycin tablets (0.25 g×2 po. Qd), flucytosine (2.5 g iv drip q12h) and fluconazole (0.6 g iv drip Qd). After treatment, the patient felt better than before with a mild headache. ADL scores were 40 before and 45 after the second surgery. An additional head CT on Feb. 22 revealed that the left subfalcine herniation was slightly relieved (Fig. 1B7). The patient was transferred to another local hospital on Feb. 22 for continuous treatment with sulfamethoxazole (po), azithromycin (po) and fluconazole (iv drip).

The patient responded favorably to the first surgery in part as showed in head CT (Fig. 1A1-7) and MRI (Fig. 2A1-4); the lesions of the right parietal lobe were obviously getting smaller. He responded well slightly to the second excision with the left occipital lobe lesions and the subfalcine herniation was getting better in head CT (Fig. 1B1-7) and MRI (Fig. 2B1-3). It is interesting that calcification was seen in the left parietal lobe and basal ganglia after Dec. 18 (Fig. 1B2), as well as the right parietal lobe after Feb. 10 (Fig. 1A6).

At the follow-up by phone, the patient was discharged from the local hospital on April 2, 2021 and showed good recovery with a ADL score of 95, in comparison to past scores of 70 (before the first surgery) and 40 (before the second surgery) as shown in Table 1. At the follow-up by phone on June. 2, 2021, the patient has been taking with sulfamethoxazole (po) and azithromycin (po) and shows slow recovery with a current ADL score of 100 as shown in Table 1.

## Discussion and conclusions

To date, over 200 cases of GAE caused by *B. mandrillaris* have been described worldwide. During 1974–2016, 109 case reports of Balamuthia disease were recorded in the United States. Most (99%) had encephalitis [4]. In China, there have been 28 cases of *B. mandrillaris* infection during the past 20 years and only 16 cases (57%) developed amoebic encephalitis [5]. Most Balamuthia cases were reported in the western and southern regions of the United States, probably because of the hot and dry environment [4]. Interestingly, this patient has been living in Southern China where it is hot and humid.

The GAE has been described mostly in immunocompromised patients, rarely in an immunocompetent host. Unlike the other amoeba species, *B. mandrillaris* affects both immunocompromised and immunocompetent people [6]. This patient has been healthy. From the presence of *B. mandrillaris* in serum, CSF, and brain tissue, he was probably infected with *B. mandrillaris* through a cutaneous lesion by contacting with the contaminated soil or water.

GAE is always fatal because of delayed diagnosis. In the beginning, this patient was considered to have a brain tumor because of unspecific symptoms and an absence of skin lesions. The progressive neurological decline after the first surgery of this patient raised the attention of neurologists with intracranial infections. Many cases of GAE were diagnosed via autopsy postmortem, sections of the skin, or brain tissue before [4]. However, with the development of NGS for pathogen detection, the diagnosis of GAE is determined in earlier stages of the disease than before. To the best of our knowledge, this is the third case of GAE reported within China via next-generation sequencing [7, 8]. As a rapid and accurate method of pathogen identification, NGS has large potential in the diagnosis of difficult and rare cases. It also has guiding significance for clinicians on deciding the treatment strategy for patients.

The combination drugs of pentamidine, sulfadiazine, flucytosine, fluconazole, azithromycin or clarithromycin, and miltefosine are recommended for treatment of Balamuthia infection in the United States [4]. In China, Lincomycin, azithromycin, interferon-γ, or a combination of these drugs showed effect in treating skin lesions, and one survivor who developed focal encephalitis was treated by excision of the infected brain tissue [5]. The 14 cases with BAE who treated with surgery and medical treatment are summarized in Tables 1, 2 [2, 9–17]. Among 14 cases, 4 patients including one child and two of old age (21.4%) responded favorably to the combined therapy with complete recovery. The neurological symptoms of the 3 patients were relatively mild and they were likely to be in the early stage of the disease. The role of surgery in Balamuthia infection is previously applied to diagnostic biopsy. It is now suggested that early surgical resection of intracranial lesions combined with drug therapy may be an important method for the treatment of Balamuthia infection. However, due to the limited number of surgical survivors, prognostic factors and effective treatment options need to be further studied.

**Table 1:** Comparison of activity of daily living after treatments with excisions and medications

Item	Before the 1st operation (Nov.9,2020)	After the 1st operation (Nov.20, 2020)	Before the 2nd operation (Dec.10, 2020)	After the 2nd operation (Feb. 8,2021)	Discharged from local hospital (Apr.2, 2021)	Stayed at home (June.2, 2021)
Defecate	Independent	Independent	Independent	Independent	Independent	Independent
Urination	Independent	Independent	Independent	Independent	Independent	Independent
Grooming	Needs help	Needs help	Needs help	Needs help	Independent	Independent
Toileting	Independent	Need some help	Dependent	Dependent	Independent	Independent
Eating	Independent	Independent	Needs some help	Independent	Independent	Independent
Transferring	Independent	Independent	Needs some help	Needs a lot of help	Independent	Independent
Activities	Needs some help	Independent	Needs some help	Needs some help	Independent	Independent
Dressing	Needs some help	Needs some help	Dependent	Needs some help	Independent	Independent
Climbing Stairs	Needs help	Needs help	N/A (stayed in bed)	N/A (stayed in bed)	Independent	Independent
Bathing	Dependent	Dependent	Dependent	Dependent	Dependent	Independent
Score	70 points	75points	40 points	45 points	95points	100points

**Table 2:** 14 cases with Balamuthia amoebic encephalitis treated with surgery and medications

Patient	Region	Age	Sex	Neurological symptom and sign	Medical treatment	Outcome	References
1	Arizona	13	Female	Left hemiparesis, slurred speech, headaches, emesis	Dexamethasone, levetiracetam, vancomycin, ceftriaxone, metronidazole, miltefosine, fluconazole, flucytosine, azithromycin, sulfadiazine	Death	[2]
2	China	7	Female	Red plaque on the face	Lincomycin, interferong-γ	Complete recovery for 13 years	[5]
3	Thailand	4	Female	Headaches, vomiting, ataxia, cerebellar nystagmus	Pentamidine, sulfasalazine, fluconazole, clarithromycin, amphotericin B	Death	[9]
4	Texas	38	Male	Seizures, headaches, lethargy, left hemiparesis	Phenytoin, prednisone, broad spectrum antibiotics	Death	[10]
5	Japan	68	Male	Seizures, right hemiparesis, unconscious	Prednisone, levetiracetam	Death	[11]
6	Australia	80	Female	Generalized seizures	Prednisolone, ceftriaxone, penicillinG, metronidazole, azithromyci, itraconazole, sulfadiazine, flucytosine, liposomal amphotericin	Complete recovery for 18 months	[12]
7	Japan	57	Female	Mild dysarthria, fever, headaches, vomiting, coma	Cefazolin, dexamethasone, ceftriaxone, metronidazole, sulfamethoxazole, trimethoprim, meropenem	Death	[13]
8	Argentina	12	Male	Fever, lethargy, right hemiparesis	Prednisone, pentamidine, fluorocytosine, fluconazole	Death	[14]
9	Argentina	5	Male	Seizures, unconscious	Pentamidine, 5-fluorocytosine, fluconazole, clarithromycin	Death	[14]
10	Argentina	3	Female	Seizures, left hemiparesis, coma	Pentamidine, 5-fluorocytosine, fluconazole, clarithromycin	Death	[14]
11	Argentina	6	Male	Right hemiparesis, unconscious	Pentamidine, 5-fluorocytosine, fluconazole, clarithromycin	Death	[14]
12	Portugal	8	Male	Headaches, vomiting, lethargy, medial esotropia	Fluconazole, rifampin, trimethoprim-sulfamethoxazole	Death	[15]
13	Texas	69	Female	Altered mental, ataxia, cranial nerve palsy	Sulfadiazine, zithromycin, fluconazole miltefosine, pentamidine	Death	[16]
14	California	84	Male	Altered mental, headaches	Doxycycline, minocycline, topical clobetasol, azithromycin, flucytosine, sulfadiazine	Complete recovery for 1 year	[17]

In conclusion, this is the first report of an immunocompetent patient with GAE caused by *B. mandrillaris* survived with two excisions and medications. Early surgical resection of intracranial lesions combined with medication treatment may offer the chance of a cure.

## Supplementary Information

**Additional file 1. **CARE Checklist of information to include when writing a case report

## Data Availability

All data generated or analyzed during this study are included in this article.

## Abbreviations

GAE: Granulomatous amoebic encephalitis
NGS: Next-generation sequencing
CSF: Cerebral spinal fluid
BAE: *B. mandrillaris* encephalitis
MRI: Magnetic resonance imaging
CT: Computed tomography
ADL: The activity of daily living
ESR: Erythrocyte sedimentation rate
CRP: Hypersensitive C-reactive protein
